# Initial Observation of Factors Interfering with the Treatment of Alveolar Osteitis Using Hyaluronic Acid with Octenidine—A Series of Case Reports

**DOI:** 10.3390/biom11081157

**Published:** 2021-08-04

**Authors:** Martin Kapitán, Jan Schmidt, Radovan Mottl, Nela Pilbauerová

**Affiliations:** Department of Dentistry, Charles University, Faculty of Medicine in Hradec Králové and University Hospital Hradec Králové, Sokolská 581, 50005 Hradec Králové, Czech Republic; KapitanM@lfhk.cuni.cz (M.K.); radovan.mottl@fnhk.cz (R.M.); nela.pilbauerova@lfhk.cuni.cz (N.P.)

**Keywords:** Alveolar osteitis, hyaluronic acid, octenidine, risk factors, smoking, treatment effectivity, wound healing

## Abstract

Alveolar osteitis (AO) is a common complication following the extraction of the teeth, particularly the lower third molars. It starts within a few days after the extraction and manifests mainly as pain in the extraction site. Several strategies of treatment are available in order to relieve pain and heal the extraction wound. Recently, a novel medical device combining hyaluronic acid (HA) and octenidine (OCT) was introduced for the treatment of AO. This series of case reports aims to summarize the initial clinical experiences with this new device and to highlight factors possibly interfering with this treatment. The medical documentation of five patients with similar initial situations treated for AO with HA + OCT device was analyzed in detail. Smoking and previous treatment with Alveogyl (Septodont, Saint-Maur-des-Fossés, France) were identified as factors interfering with the AO treatment with the HA + OCT device. In three patients without these risk factors, the treatment led to recovery within two or three days. The patient pretreated with Alveogyl and the smoker required six and seven applications of the HA + OCT device, respectively. According to these initial observations, it seems smoking and previous treatment with Alveogyl prolong the treatment of AO using the HA + OCT device that, in turn, shows a rapid effect if these risk factors are not present.

## 1. Introduction

One of the common complications of tooth extraction is alveolar osteitis (AO; dry socket). It usually starts within a few days following the extraction [1]. The blood clot in the extraction wound disintegrates, which exposes the socket walls and leads to bone inflammation. Patients suffer from a sharp pain in and around the extraction site, which may radiate to the mandibular ramus and the temporal area [2]. Other symptoms, such as trismus, fetor ex ore, and cheek swelling, could be present as well [3,4]. There are several risk factors of AO, including traumatic surgery, transalveolar extraction, acute pericoronitis, smoking, and oral contraceptives [3,5,6,7,8,9,10]. The incidence of AO varies between 0.5% and 5% for a routine tooth extraction but is up to 37.5% for the mandibular third molar extraction [6].

During the examination of the patient, it is necessary to exclude the presence of a foreign body in the extraction wound, i.e., a fragment of the tooth or bone, filling particle, etc. The treatment always starts with thorough wound debridement and irrigation (hydrogen peroxide or chlorhexidine). Subsequently, the local intervention continues with the goal to relieve pain and heal the extraction wound. Several different methods are recommended, such as Zinc oxide eugenol (ZOE), Alveogyl, vitamin C, plasma rich in growth factors, local anesthetics, topical antibiotics, or low-level laser application [7,9,11,12,13]. The standard treatment method used in the authors’ workplace is the short-term intra-appointment placement of gauze soaked with a phenol-camphora spirituous solution in the extraction wound combined with the application of dry heat (infrared lamp) for 15 min, which brings an immediate pain relief in the majority of the patients [2]. After removal of the gauze, the extraction wound is covered with ZOE paste or filled with Alveogyl, consisting of eugenol and Penghawar Djambi [14]. The advantages are analgesia and disinfection; however, it presents several risks, such as allergic reaction, prolonged healing, and chronic inflammation. The fibers of Penghwar Djambi, even though expected to be absorbable, persist in the extraction wound and can complicate further implantation [9]. Systemic antibiotics are not recommended for the risk of an allergic reaction and contribution to the antimicrobial resistance, which overrides the treatment benefit. Patients with compromised immune functions or the signs of inflammation spreading to the surrounding regions are a rare exception.

A new remedy based on hyaluronic acid (HA) and octenidine (OCT) is currently in Phase II of a clinical trial. It was developed for the treatment and prevention of AO [15].

Hyaluronic acid is a glycosaminoglycan biopolymer consisting of disaccharide units (d-glucuronic acid and d-*N*-acetylglucosamine). Its long chains provide elasticity and support cell migration and proliferation within most of the tissues. HA splits in chains of low molecular weight in case of tissue damage. These fragments then initiate the early stage of the inflammatory reaction [16,17]. Later, when granulation tissue is formed, HA absorbs free radicals and reduces oxidative stress in the newly formed tissue [18,19]. HA is biodegradable and, as a ubiquitous component of tissues, biocompatible.

Octenidine dihydrochloride (*N*,*N*’-(1,10-decanediyldi-1[4H]-pyridinyl-4-ylidene)bis(1-octanamine) dihydrochloride) is a cationic surface-active disinfectant with wide antimicrobial, antifungal, and antiviral effects. It is broadly used on skin and mucosa [20]. Its non-specific mechanism of action is not expected to induce microbial resistance, which benefits OCT in comparison to antibiotics [21]. Other advantages are prolonged effects due to the temporary binding to the mucosa surface and no systemic adverse effects [22]. It can be used in children and pregnant women [23].

**Figure 1:**
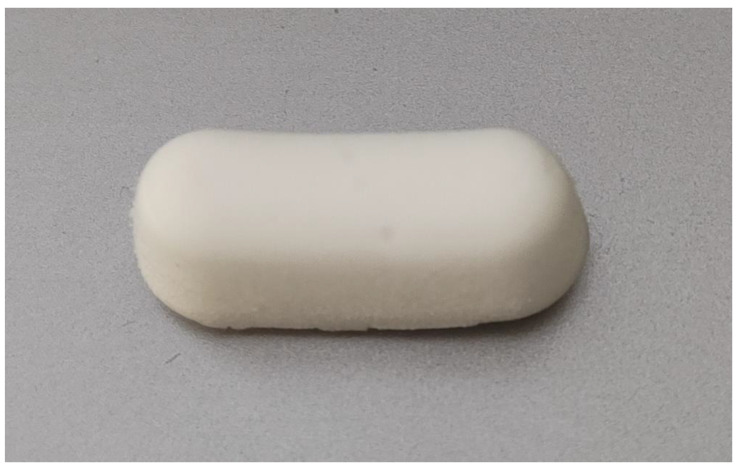
The HA + OCT device has a shape of a cuboid with round edges, white color, sponge-like consistency, and size of 20 mm × 5 mm × 5 mm.

Suchánek et al. [15] recently introduced a new treatment protocol of AO. The principle is the placement of the HA + OCT device (Figure 1) in the extraction wound after its irrigation with 2 mL of 3% hydrogen peroxide and 2 mL of water for injection. This is done on a daily basis until full relief, or for a maximum of 7 days. In the study, the visual analogue scale (VAS) was used to evaluate pain; VAS < 20/100 was considered a threshold of the full relief. The treatment outcome was favorable in the majority of the patients included in the first clinical study, with the median number of applications being four [24].

This case report series aims to demonstrate the progress of healing of AO using the treatment protocol mentioned above and to point out several factors possibly interfering with the effect of such treatment.

## 2. Materials and Methods

Twenty-three patients from the multi-center, open-label, first clinical study (the Czech Republic State Institute for Drug Control ref. sukl122215/2015, Clinicaltrial.gov ID: NCT04091399) were analyzed. The study was approved by the Ethics Committee of the University Hospital Hradec Králové on 6 March 2015, ref. 201503 D02ZP. The medical records of the patients treated in one of the involved centers (The Department of Dentistry, Charles University, Faculty of Medicine in Hradec Králové, and University hospital Hradec Králové) were studied to find factors eventually influencing the effect and progress of the treatment. Smoking and previous AO treatment with Alveogyl came to our attention due to the prolonged AO healing period. Five patients with a similar initial situation were selected for a demonstration of different postoperative courses according to whether these factors were present or not.

## 3. Results

### 3.1. General Findings

All the selected patients had undergone a transalveolar extraction of the mandibular left third molar (tooth No. 38 in *Fédération Dentaire Internationale* (FDI) system); the mandibular block was used as local anesthesia. The extractions were performed at our department in the first four patients, while in the fifth patient, the tooth was extracted elsewhere. AO followed the extractions, and the patients came to our department to seek aid. Coincidentally, all of them were women. They were treated using the new medical device based on HA and OCT. Two patients without the risk factors recovered quickly after only two and three applications, respectively. The third patient was without risk factors, but the dry heat was applied in combination with the HA + OCT device, which led to a collapse of the patient; then, the treatment lasted 3 days. The fourth patient (pretreated with Alveogyl) needed six applications; the fifth patient (a smoker of 10 cigarettes a day) required seven visits until VAS decreased under desired 20/100. A detailed description of the cases follows. As mentioned above, the extracted teeth were identical, and the workplace was the same for all the presented patients. Thus, these details will not be repeated on a case-by-case basis.

### 3.2. Case Report 1

**Figure 2:**
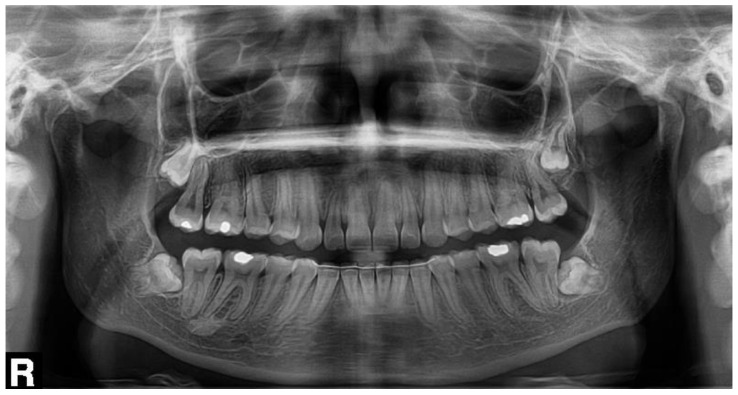
The orthopantomogram of the patient 1. Tooth No. 38 is semi-impacted in a mesially inclined, almost horizontal position. There is a burnout around the crown of the tooth.

An 18-year-old female patient underwent a transalveolar extraction of an impacted tooth No. 38. She declared increased stomach pH, but no other diseases, no permanent medication, no allergies, and she smoked five cigarettes a day. The reason for extraction was repeated pain and pressure in the bone in the area of this tooth and a burnout around the tooth crown visible in the orthopantomogram (OPG) (Figure 2). Before the extraction, there was no pain nor swelling. According to the surgeon, the extraction was difficult and took approximately 30 min. Peroral antibiotics were prescribed—phenoxymethylpenicillin 1.5 million international units (MIU) t.i.d.

Five days after the extraction, severe pain in the extraction site started, irradiating to the left ear. She visited our department on the following day (six days after the extraction). She had to take peroral analgetic—nimesulid. The entry VAS was 59/100. There were food remnants in the extraction wound, and the surrounding mucosa was painful upon palpation. The suture remained in situ. Extraorally, a swelling of 2 cm × 2 cm at the mandibular angle was observed. Without local anesthesia, the wound was rinsed with 3% hydrogen peroxide, water for injection (Bieffe Medital, Grosotto, Italy), and the HA + OCT device was inserted. The patient was instructed not to smoke.

The first check-up was on the next day. The patient avoided smoking and declared complete relief after the treatment. However, in the morning, the pain started again. She did not have to take analgesics, and VAS was 54/100. The swelling was reduced to the size of 1 cm × 1 cm. The local treatment was repeated (the second application of the HA + OCT device).

Until the second check-up on the next day, VAS decreased to 2/100, and the treatment was finished.

The patient declared she had suffered from a similar problem in the past after the extraction of the mandibular right third molar (tooth No. 48 in FDI). Then, she was treated by repeated local procedure standardly used at our workplace, i.e., debridement and rinse of the extraction wound with 3% hydrogen peroxide, temporary intra-appointment placement of gauze soaked in a phenol-camphora spirituous solution in the extraction wound combined with the application of dry heat (infrared lamp) for 15 min, and insertion of Alveogyl. She found this new treatment method much better for its more rapid pain relief and absence of poor taste in the mouth.

### 3.3. Case Report 2

**Figure 3:**
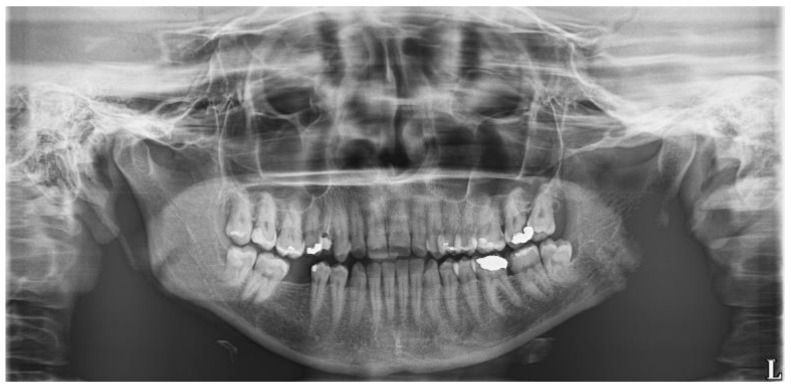
The orthopantomogram of the patient 2. Tooth No. 38 is in normal position, slightly distally inclined, with a semilunar burnout distally. The OPG was not taken at our department.

A woman of age 48 years had her semi-impacted tooth No. 38 extracted for recurrent pericoronitis. The OPG (Figure 3) showed a semilunar burnout distally from the tooth crown. The patient did not have any general disease, medication, or allergy, and she did not smoke. There was no pain nor swelling prior to the transalveolar extraction, which lasted 10 min. No antibiotics were prescribed.

Two days after the extraction, she started to feel pain in the extraction wound. Three days later (five days after the extraction), she sought aid at our department. She declared a cruel pain in the wound irradiating to the left ear and left temporal region. The patient took 1 tablet of ibuprofen 400 mg, and the entry VAS was 94/100. There was no extraoral swelling, the wound was empty with exposed bone walls, but the surrounding mucosa was not painful. Without local anesthesia, the wound was rinsed with 3% hydrogen peroxide, water for injection, and the HA + OCT device was inserted.

During the first check-up on the following day, she declared pain irradiating to the left ear, but she did not use any painkillers; VAS was 63/100. The local treatment was repeated.

In the second check-up the next day, she complained of pain during the night with VAS 32/100. The local treatment was repeated (third application of the HA + OCT device).

Until the third check-up, VAS decreased to 8/100, and the treatment was finished.

### 3.4. Case Report 3

**Figure 4:**
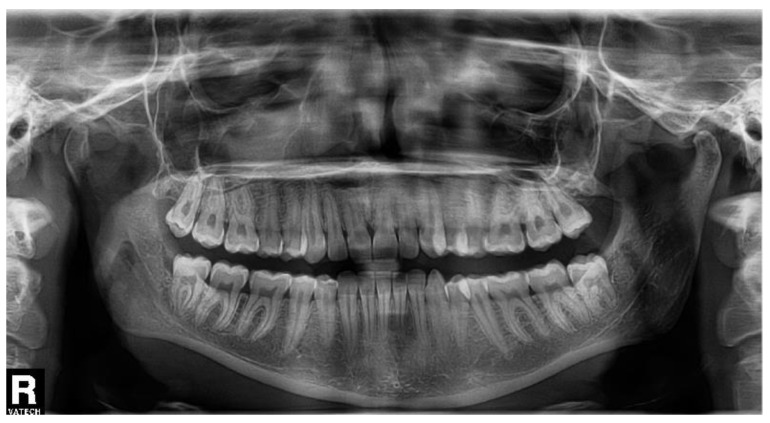
The orthopantomogram of the patient 3. Tooth No. 38 is in normal position, with a semilunar burnout distally.

The semi-impacted tooth No. 38 was extracted for recurrent pericoronitis in the 30-year-old female patient, with no general diseases, no medication, and no allergies, who was a non-smoker. The OPG (Figure 4) showed a normal position of the tooth with a semilunar burnout distally. The transalveolar extraction took 30 min. No pain or swelling was present before the surgery.

Pain in the extraction wound started two days after the extraction and brought her to our department two other days later (four days after the extraction). The patient declared dull pain spreading to the left ear and left temporal region, waking her during the night. She took three tablets of tiaprofenic acid á 300 mg, and the entry VAS was 65/100. The face was symmetric, with no swelling, the extraction wound was filled with debris and food particles, and the surrounding mucosa was not painful. Without local anesthesia, the wound was rinsed with 3% hydrogen peroxide, water for injection, and the HA + OCT device was inserted. Dry heat (infrared lamp) was applied on the left cheek for 15 min in a sitting position. During this time, the patient collapsed. She was laid down to the stabilized position and quickly recovered.

The next day, she declared worsening of the pain, which irradiated to the left ear and left temporal region; however, VAS was 59/100. The local treatment was repeated but without the dry heat application.

During the second check-up, VAS was 40/100, the patient did not take any analgesics, and the local treatment was repeated (third application).

Till the next day, VAS decreased to 20/100, and the treatment was finished.

### 3.5. Case Report 4

**Figure 5:**
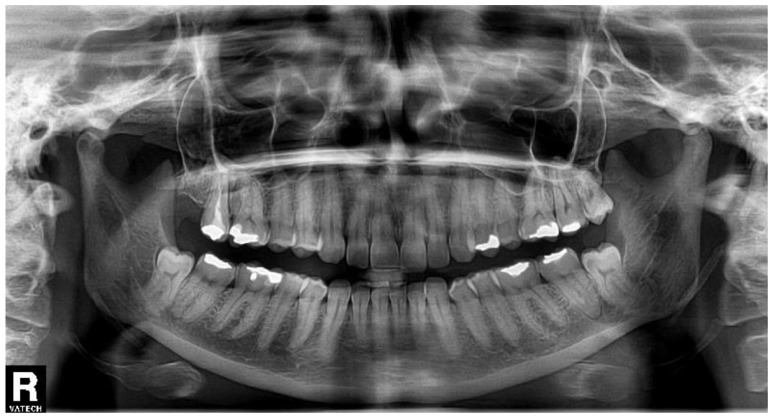
The orthopantomogram of the patient 4. Tooth No. 38 is in infra-occlusion, with a semilunar burnout distally.

A 41-year-old woman had her partially erupted tooth No. 38 extracted for repeated pain (Figure 5). She did not suffer from any general diseases, did not take any medicaments, did not smoke, and was allergic to trimethoprim/sulfamethoxazole. The transalveolar extraction took 15 min, and according to the surgeon, it was difficult. After the extraction, an i.m. injection of dexamethasone 8 mg/2 mL was applied to reduce the expected postoperative swelling. No antibiotics were prescribed.

Three days later, the pain started in the extraction wound irradiating to the left temporal area. On the following day, the patient sought aid at the dental emergency, where the extraction wound was rinsed with 3% hydrogen peroxide, and Alveogyl was placed in. Two days later, the same local treatment was performed.

The next day (seven days after the extraction), she visited our department for persisting pain in the extraction wound, with VAS 60/100. There was no extraoral swelling, and the extraction wound was empty with palpation pain of the surrounding mucosa. Without local anesthesia, the wound was rinsed with 3% hydrogen peroxide, water for injection, and the HA + OCT device was inserted.

The same local treatment was repeated daily for the following five days. The pain was mainly during night, and VAS slowly decreased through 37/100, 34/100, 31/100, 27/100, and 26/100, to the final 10/100 in the sixth check-up, when the treatment was finished.

### 3.6. Case Report 5

A 28-year-old female patient underwent a transalveolar extraction of the tooth No. 38 at another workplace for repeated pain in that area. The patient used antidepressive drugs, declared no allergies, and smoked 10 cigarettes a day. The extraction lasted for 45 min, according to the patient.

**Figure 6:**
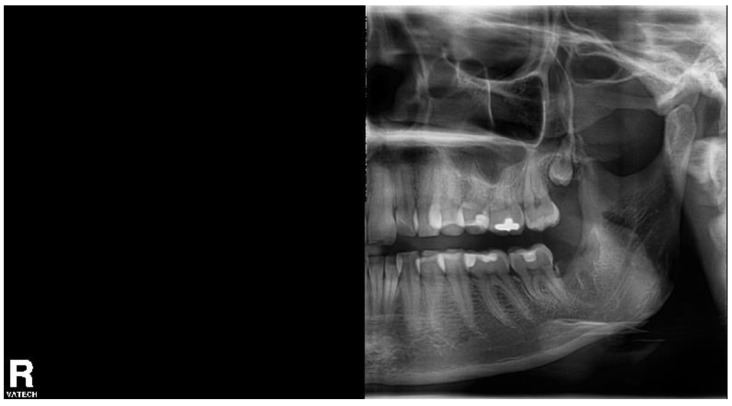
The half orthopantomogram of the left side of the patient 5. An empty socket of the tooth No. 38 is visible.

The pain started immediately after extraction, the patient used painkillers (the drug name was not specified) with no significant effect, and four days later, she came to our department. She complained of pain in the extraction wound, VAS 38/100. There was no extraoral swelling, the range of mouth opening was limited to 2.5 cm, the extraction wound was filled with detritus, and the surrounding mucosa was painful upon palpation. A half OPG was taken to confirm no broken root in the socket (Figure 6). Without local anesthesia, the wound was rinsed with 3% hydrogen peroxide, water for injection, and the HA + OCT device was inserted.

The local treatment was repeated during the following days, while the patient continued smoking 5–10 cigarettes a day. Six more applications of the HA + OCT device were needed until the pain (mainly during the night) was relieved. VAS was subsequently 35/100, 43/100, 32/100, 30/100, 29/100, and 27/100. Finally, on the seventh check-up, the patient was completely pain-free, and the treatment was finished.

## 4. Discussion

This series of case reports presents the effect of the AO treatment with a novel medical device based on HA and OCT. The conclusions are limited by a small number of observed and described patients. However, general tendencies can be seen. Some of the possible biases are eliminated by comparing cases of the same starting situation (AO after transalveolar extraction of tooth No. 38—FDI) and the same gender.

Out of 23 patients treated for AO in our department within this study, there were 14 women (61%). This is in accordance with the scientific literature, which mostly agrees with the higher incidence of AO among female patients [25,26,27]. Additionally, the role of oral contraceptives and the menstruation cycle phase at the time of extraction has been proved [6,27,28].

We observed relatively rapid healing in the first two patients without any apparent risk factors. This treatment of AO with a new HA + OCT device seems effective, and the healing is faster or similar (2–8 days, median 4 days [15,24]) to other commonly used treatment modalities (3–10 days [7,11,13]).

The eugenol-based agents (Alvogyl and Alveogyl, Septodont, Saint-Maur-des-Fossés, France) are popular for their proven effectivity in pain reduction [6,7,12], although eugenol causes prolonged healing of the wound [29]. All previous studies were done with Alvogyl, which contained butamen, iodoform, and eugenol. This medicament was replaced with Alveogyl with a different composition—eugenol and Penghawar Djambi [14]. It seems a significant proportion of dental professionals remains unaware of the change of both the name and the composition [14]. Another disadvantage is the non-resorbability of the material [9,29], which, in the case of improper removal, can lead to complications during the further implantation, as the material stays unresorbed in situ.

Other treatment methods (local anesthetics, topical antibiotics, low-level laser, plasma rich in growth factors) seem to be similarly or less effective, less commonly used, or less available in the general dental offices [6,7,9,12,13].

The dry heat application without the simultaneous placement of anti-inflammatory agents into the extraction wound might have caused the third patient’s collapse during the entry visit. The standard treatment in our department includes the placement of gauze soaked with a phenol-camphora spirituous solution in the wound during the heat application. In this patient, dry heat was applied in combination with the HA + OCT device, i.e., without any anti-inflammatory effect. This could have accelerated the acute inflammation of the bony socket walls resulting in a collapse of the patient. After that, the overall time of treatment was three days, as there were no contributing risk factors.

The fourth patient was pretreated with Alveogyl placed twice in the extraction wound before the HA + OCT treatment with the device was initiated. The effect of eugenol on prolonging the healing probably persisted and caused the need for a total of six applications of the HA + OCT device. This finding indicates a history of Alveogyl application lowers the effectiveness of HA + OCT treatment. Thus, this combination shall be avoided. However, further investigation is needed as this conclusion must be assessed on a larger data set.

The fifth patient was a smoker, and although she was instructed not to smoke at least during the treatment of AO, she continued to do so. Smoking is a risk factor for the AO onset [6,8,10] and contributes to prolonged healing. The effect of smoking is mainly in vasoconstriction of blood vessels in the mucosa and local affection of inflammatory reaction, which leads to prolonged healing of mucosa wounds in general [7,30]. Additionally, the sucking action during the smoking may play a role in the disintegration of the blood clot [6,26]. It is likely that smoking is another factor hindering the AO treatment with the HA + OCT device, similarly to other treatment modalities. Even though patient No. 1 was also a smoker, the response to the treatment was good because she avoided smoking during the treatment of AO.

According to these preliminary observations, several factors can decrease the effectiveness of the new HA + OCT device in the treatment of AO, namely smoking and previous treatment with Alveogyl. The limitation is the small number of the involved patients. Further studies including more significant cohorts of patients are necessary for confirmation and discovery of other aspects affecting the treatment after the device finishes the registration process.

## 5. Conclusions

- The treatment of AO using HA + OCT is fast, effective, and simple.
- Smoking and previous application of Alveogyl seem to interfere with the effect of this treatment.

## Data Availability

Data sharing is not applicable to this article.
